# Energy-efficient population coding constrains network size of a neuronal array system

**DOI:** 10.1038/srep19369

**Published:** 2016-01-19

**Authors:** Lianchun Yu, Chi Zhang, Liwei Liu, Yuguo Yu

**Affiliations:** 1 Institute of Theoretical Physics, Key Laboratory for Magnetism and Magnetic Materials of the Ministry of Education, Lanzhou University, Lanzhou, 730000, China; 2 State Key Laboratory of Theoretical Physics, Institute of Theoretical Physics, Chinese Academy of Sciences, Beijing, 100109, China; 3 Cuiying Honors College, Lanzhou University, Lanzhou, 730000, China; 4 College of Electrical Engineering, Northwest University for Nationalities, Lanzhou, 730070, China; 5 School of Life Science and the Collaborative Innovation Center for Brain Science, Center for Computational Systems Biology, Fudan University, 200433, China

## Abstract

We consider the open issue of how the energy efficiency of the neural information transmission process, in a general neuronal array, constrains the network size, and how well this network size ensures the reliable transmission of neural information in a noisy environment. By direct mathematical analysis, we have obtained general solutions proving that there exists an optimal number of neurons in the network, where the average coding energy cost (defined as energy consumption divided by mutual information) per neuron passes through a global minimum for both subthreshold and superthreshold signals. With increases in background noise intensity, the optimal neuronal number decreases for subthreshold signals and increases for suprathreshold signals. The existence of an optimal number of neurons in an array network reveals a general rule for population coding that states that the neuronal number should be large enough to ensure reliable information transmission that is robust to the noisy environment but small enough to minimize energy cost.

Neuronal activity, related to information processing in brain circuits, is metabolically expensive^1^. For example, the metabolic cost of the human brain may rise from 20% to 40% of whole-body energy production when human beings switch from a resting state to a working state. Action potentials, which are electrical signals and rely on the potential energy stored in transmembrane ion gradients, cost a large fraction of this energy^2^. These metabolic demands could be large enough to influence the design, function and evolution of brains^3^,^4^,^5^,^6^,^7^,^8^,^9^,^10^.

Population coding^11^, i.e., the cooperative coding of information of input signals by a group of neurons, is a basic neural code strategy used in many nervous systems^12^. Studies have shown that neuronal activities could be synchronized to remain robust against noise and promote the reliable transmission of information^13^,^14^,^15^. It was suggested^16^,^17^ that the number of neurons involved in synchronous neuronal activities is critical for reliable information delivery within a feed-forward multi-layer cortical network. However, for population coding in such an array network, the relationship between efficient energy consumption to information transmission and neuronal number has not been carefully considered, especially in the case of different background noise levels.

Moreover, background noise is present at all levels of the nervous system, from the microscopic level, such as stochastic ion channel gating in membranes and biochemical noise at synapses, to macroscopic levels^18^,^19^,^20^. The existence of noise may degrade the reliability of effective information transmission and requires the involvement of more neurons to perform an information-processing task^15^. Therefore, it is critical to address the issue of what is the proper size of a neuronal array network for reliable information transmission with minimal energy cost in a noisy environment.

In an earlier work, Barlow (1961)^21^ suggested sparseness as one of the principles that are important to sensory representation. Because sparse codes are defined as representations with low activity ratios—i.e., at any given time a small proportion of neurons are active—they are sometimes proposed as a means to help conserve metabolic costs. Levy and Baxter (1996)^22^ demonstrated that there should exist an optimal firing probability for any given mean firing rate in a neural network so that the capacity to represent sensory information is maximized while energy expenditure is minimized. Later, a quantitative measure of the metabolic cost of neural electrical signals generated by retina photoreceptors^3^ and action potential generation in axons was estimated by experimental as well as computational studies^5^,^7^,^8^,^9^,^10^,^23^,^24^,^25^. These studies have linked the energy efficiency of spiking with the number of the ion channels^19^, as well as the ratio of sodium to potassium channel density within a single neuron^5^,^7^,^9^,^23^,^24^,^25^. However, the way limited energy constrains the size and coding efficiency of a neuronal network, in the presence of different noise levels, has not been carefully examined. In the past few decades, a number of studies of stochastic resonance have shown that noise plays an important role in neural information processing for sub- or suprathreshold signals^14^,^26^,^27^. In this paper we have carried out a mathematical analysis and numerical simulations to investigate the issue of network energy coding efficiency and its dependence on the population size, and signal and noise intensity. We have considered a neuronal network with a type of excitable neuronal model.

We proceeded by first solving a stochastic one-dimensional bistable Langevin equation, which mimics action potential generation with a particle crossing the barrier of a double well, to obtain an analytical solution for the pulse signal detection rate and spontaneous firing rate^28^. Coincidence detector (CD)^29^,^30^,^31^,^32^ in the context of neurobiology is a process by which a neuron or a neural circuit can encode information by detecting the occurrence of temporally close but spatially distributed input signals from presynaptic neurons of a network. A number of reports^31^,^32^,^33^ have suggested that neuronal network with postsynaptic CD might be commonly used in different cortical areas to read synchronous activities against a noisy background. Here, we have constructed an array network model with *N* bistable neurons and a CD neuron to pool the network information, and calculated the mutual information and energy cost of the network.

## Results

The bistable neuron model used here can be described with the following equation:


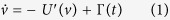


where *v* is the membrane potential, and *U* is a double well potential, defined as:


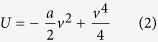


Note that *U* has two minima at 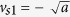, 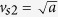 and a saddle point at 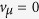. In the following calculation, 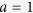 by default. 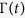 is a Gaussian white noise, with


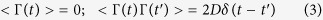


and D the noise intensity. We assume the neuron to be in its resting state when the particle is in the left well and in the excited when the particle has crossed the barrier to the right well, due to noise or signal stimulation.

It is assumed that a force of short time duration moves the particle horizontally from the resting state to *v*′ in the region of the saddle point. When the force disappears, the particle drifts up to the region of the saddle point. Near the saddle point, the phase trajectories are repelled, causing the particle to accelerate away from the saddle-point region towards one of the two minima. According to Lecar and Nossal’s approach of linearizing around the saddle point, we can obtain the probability of finding the particle in the right well after a long enough time, i.e., the probability that a pulse input signal is detected by the neuron^20^,^34^,


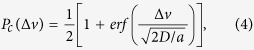


where 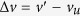 is the strength of the pulse inputs, and 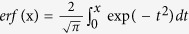 is the Gaussian error function. With no input signal, neurons fire spikes spontaneously under perturbation by noise. The spontaneous firing rate of a bistable neuron is derived by Kramers’ formula^35^:


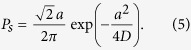


**Figure 1:**
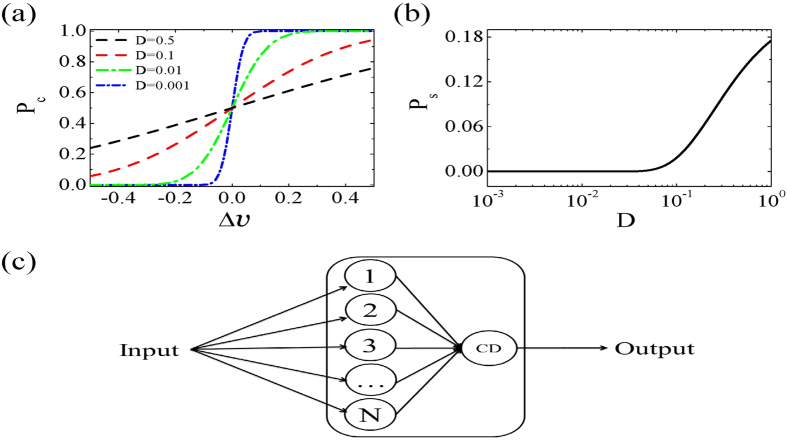
(**a**) The detection rate of the bistable neuron model as a function of input pulse strength for different noise intensities. (**b**) The bistable neuron model’s spontaneous firing rate as a function of noise intensity (**b**). (**c**) The network model with an array of N neurons and a coincidence detector (CD) neuron with a spiking threshold 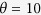.

Figure 1(a) shows the firing probability of the neuron as a function of input pulse strength Δ*v* with different noise intensities D, described by Eq.4. It is clear that the firing threshold fluctuates depending on the strength of noise perturbation. The neuron fires in response to subthreshold inputs (Δ*v* < 0) with the assistance of noise, which is well known as stochastic resonance^28^. The detection rate increases as the noise intensity increases. However, the noise sabotages the neuron’s reliability for suprathreshold inputs (Δ*v* > 0), and the detection rate decreases as the noise intensity increases. For threshold inputs (Δ*v* = 0), the detection rate is 50%, independent of noise intensity. Our results are consistent with previous simulation results for a Hodgkin-Huxley (HH) system (for example, see reference^36^ where the inputs are in the form of voltage pulses). Figure 1(b) shows that the spontaneous firing rate increases as a function of noise intensity. The same result was obtained by Zeng *et al.* with the stochastic simulation of the HH neuron^37^.

Next we consider the information capacity and energy cost of an array of *N* bistable neurons with a pulse signal as input whose intensity is distributed uniformly over the interval 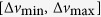, with the probability density function 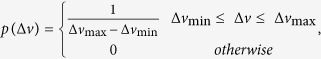 and the mean input strength 
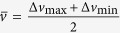
. The information in the synchronous firings of this N neuron array is pulled together by a CD neuron, see Fig. 1(c). The CD neuron receives the outputs of the *N* bistable neuron array and is excited if n (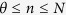) inputs arrive simultaneously, where *θ* is the threshold of the CD neuron. In response to pulse inputs, this network has two output values, i.e., *R* = {*r| r* = *1 if CD neuron fires, or = 0 if CD neuron fails to fire*}. The conditional probability 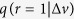 that the CD neuron fires when the input is ∆*v* is given by a cumulative binomial distribution


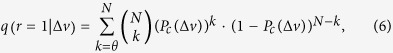


where 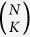 is a binomial coefficient, and 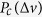 is the detection rate of the bistable neuron for pulse input with strength ∆*v*, determined by Eq. 4. The conditional probability that the CD neuron does not fire when the input is ∆*v* is given by


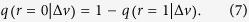


According to Bayes formula, the probability that the output is *r* can be obtained by


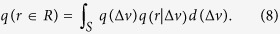


According to Shannon information theory^38^, the information between input *S* and output *R* is defined as


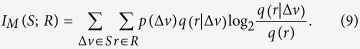


In our case, the input is continuous and the output is discrete, and thus, the summation must be rewritten as follows:


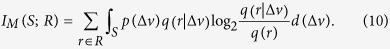


Finally, we obtain the following description of the mutual information for the CD neuron:


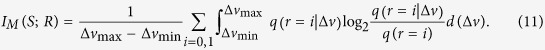


**Figure 2:**
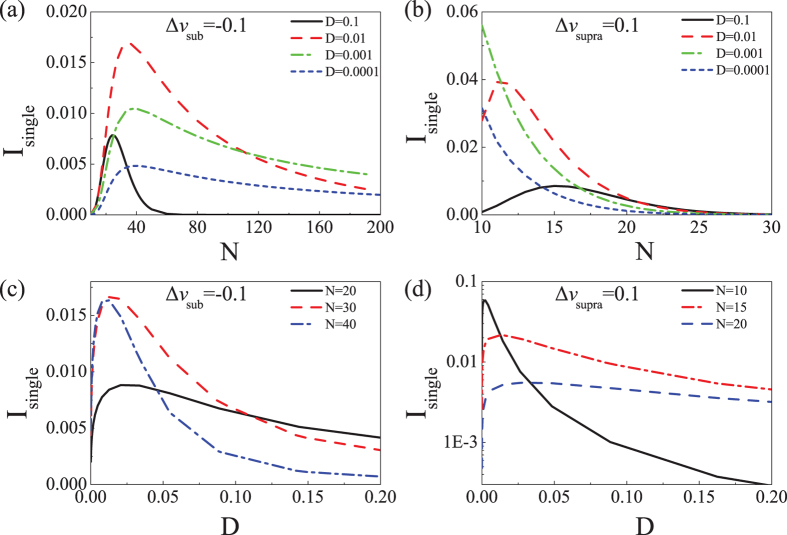
The average mutual information per neuron *I*_single_ as a function of array size N for subthreshold signal Δ*v*_sub_ = −0.1 (**a**) and for suprathreshold signal 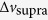 = 0.1 (**b**). (**c**) 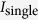 vs. noise intensity D for neuronal numbers N = 20, 30, and 40. (**d**)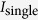 vs.D for N = 10, 15, and 20.

Figure 2(b) shows that 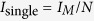, the average mutual information per single neuron can reach a global maximum when the network contains an optimal number of neurons for subthreshold signals (e.g., 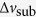 = −0.1). The optimal neuronal number becomes smaller when the noise intensity increases. For superthreshold signals (e.g., 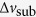 = 0.1), the average mutual information per single neuron can also be maximized by an optimal neuronal number. However, when the noise intensity decreases, the optimal neuronal number also decreases (see Fig. 2(b)). From Fig. 1(a), we see that for both subthreshold and superthreshold signals, *P*_*c*_ converges to 0.5 as the noise intensity increases. As a result, in Fig. 2(a,b), the optimal neuronal number moves to 20 with the CD threshold set to 10(results not shown). In contrast, with decreasing noise, to re-establish a maximum, the subthreshold signals require an increased number of neurons to compensate for the loss due to decreased *P*_*c*_, whereas the suprathreshold signals must decrease the number of neurons to limit the excess due to decreased *P*_*c*_. For both the subthreshold and superthreshold signals, the average mutual information per neuron can be maximized by an optimal noise intensity, displaying a classic subthreshold stochastic resonance phenomenon (see Fig. 2(c)) and suprathreshold stochastic resonance phenomenon (see Fig. 2(d))^39^.

**Figure 3:**
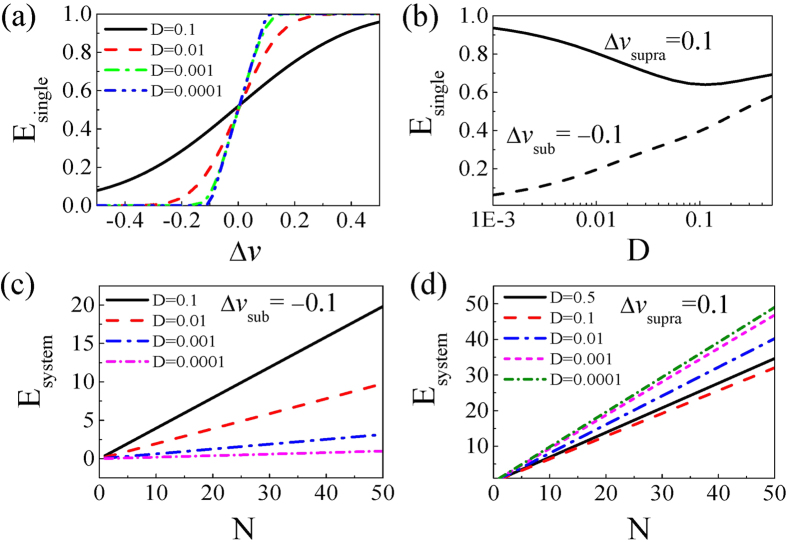
(**a**) The average energy cost per neuron *E*_single_ as a function of input pulse intensity 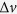 for different noise intensities D. (**b**) *E*_single_ as a function of D for 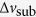 = −0.1 and 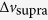 = 0.1, respectively. (**c**) The total network energy consumption *E*_system_ as a function of neuronal number N for 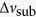 = −0.1. (**d**) *E*_system_ as a function of neuronal number N for 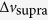 = 0.1.

For an *N* neuron array system, the total network energy expenditure in an action potential onset time interval Δ*t* can be written as


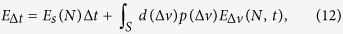


where 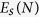 and 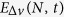 are the energy costs related to action potential generation. For simplicity, we assume the energy cost of one action potential to be 1. 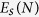 is the energy cost of the spontaneous firings in unit time, and 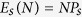. 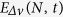 is the energy cost of the action potentials in response to input pulses with strength 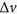, and 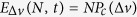 if the inputs are applied at the beginning of this time interval and zero otherwise. Therefore, 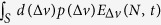 is the average energy cost of action potentials in response to an input pulse with distribution *p*(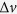). Fig. 3(a) shows the dependence of 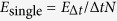, the average energy cost of each neuron in unit time, as a function of input pulse strength 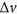 for different noise intensities. Note that when the noise is weak, as the spikes are mostly induced by the signals, the 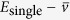 curves have similar behavior to the 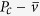 curves shown in Fig. 1(a). Interestingly, for subthreshold signals, e.g., 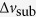 = −0.1, *E*_single_ increases as noise intensity D increases, while for superthreshold signals, e.g., 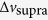 = 0.1, *E*_single_ first decreases and then increases slightly as noise intensity D increases (see Fig. 3(b)). For subthreshold signals, e.g., 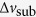 = −0.1, 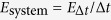, the energy consumption of the whole system in unit time, increases monotonously with both noise intensity and neuronal number; see Fig. 3(c). For superthreshold signals, e.g., 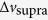 = 0.1, *E*_system_ also increases with neuronal number. However, *E*_system_ is large for weak noise intensity and becomes small for high noise intensity, see Fig. 3(d).

We now define a new measurement to quantify how efficiently the system utilizes a certain amount of energy in a certain amount of information transmission, i.e., energy cost per unit information transmission or coding energy cost: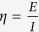, where E is the average energy consumption in unit time per neuron, and I is the average mutual information between the inputs and outputs of the neuron array in unit time per neuron. Now, we have


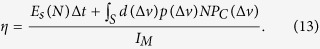


**Figure 4:**
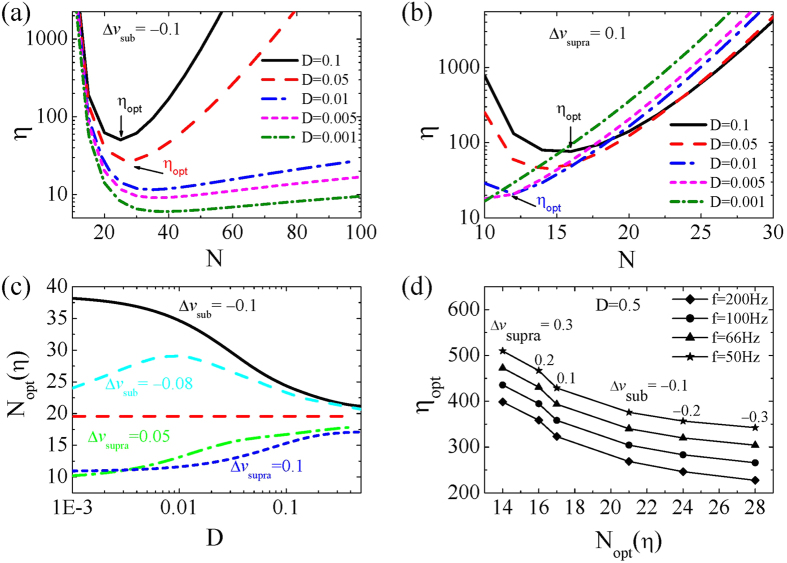
(**a**) Coding energy cost η as a function of N for 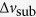 = −0.1 in the cases of different noise intensities. (**b**) η vs. N for 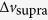 = 0.1 in the cases of different noise intensities. (**c**) The optimal neuronal number *N*_opt_ vs. noise intensity D for different input signal intensities 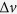. (**d**) The minimum coding energy cost *η*_opt_ vs. the optimal neuronal number *N*_opt_ for different signal intensities 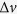 in the case of different input pulse frequencies.

Laughlin *et al.* found that in noise-limited signaling systems, a low capacity weak pathway transmits information more economically, which promotes the idea of distributed information coding among multiple pathways^3^. The analysis result for the array network supports this idea. Fig. 4(a) shows that for subthreshold signals, though the network detection rate for a single neuron is low, it yields a low coding energy cost in the information coding process for weak noise intensity. Moreover, our results show that the coding energy cost passes through a global minimum as a function of neuronal number within the network for different noise intensities. The optimal neuronal number *N*_opt_, corresponding to the minimum coding energy cost 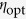, shifts to the smaller number as noise intensity increases. For a suprathreshold stimulus, the coding energy cost also passes through a global minimum as a function of neuronal number. However, the optimal neuronal number corresponding to the minimum coding energy cost shifts to the larger number side as noise intensity increases (see Fig. 4(b)). Interestingly, as the noise intensity increases, the optimal neuronal number *N*_opt_ for both sub- and suprathreshold signals converges to a small range between N = 15 and 25 (see Fig. 4(c)). This convergence occurs because the optimal neuronal number for maximal mutual information, as we discussed above (Fig. 2(a,b)), will converge from the opposite direction to 20 in the case of the large noise limit, recalling that the energy cost does not change greatly for different noise intensities. Moreover, we found that for a given noise intensity (e.g., D = 0.5), the maximal input pulse frequency that the bistable neurons can receive, which is the inverse of the action potential onset time 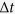, can significantly modulate the values of either *N*_opt_ or 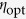 for different input pulse intensities, suggesting an energy saving mechanism for information coding in higher frequency bands, as observed in recent experimental findings^25^.

## Discussion

Consuming only several watts of energy, mammalian brains are able to carry out 1000 trillion operations per second^40^. The biophysical mechanism of this extremely efficient energy expenditure is still not fully known. In a real living brain circuit, background noise is present at all levels of the nervous system, from the microscopic level, such as channel noise in membranes and biochemical noise at synapses, to macroscopic levels, such as a small neuronal circuit composed of several to tens of neurons. The existence of noise may degrade the reliability of effective information transmission and requires the involvement of more neurons to perform an information-processing task^15^. For example, small neurons will cost less energy because fewer ion channels are involved, thus requiring less ion exchange through the ion pumps that drive ATPase Na + /K + exchanges after action potentials^9^. However, the stochastic nature of ion channel gating will not only produce variability in the response of neuron to external stimuli but also cause spontaneous action potentials, damaging the reliability of signal processing^18^. In this case, trade-offs between information transfer and energy cost may constrain the proper number of ionic channels in individual neurons^19^,^20^ as well as the proper size of neuronal number in a neuronal network. There should exist a general rule for energy consumption, neural information transmission and network size.

Earlier theoretical studies^21^,^22^ suggested that an energy efficient coding of a neuronal network should be one where a small fraction of neurons use low firing rates to encode each component of input signal, leading to the concept of sparse coding. However, our theoretical analysis suggests that involvement of background noise at different intensities should justify this sparse coding theory. Noise brings two things to the neural population coding. First, a low level of noise may deliver energy to neurons within networks to fire more electronic signals in response to input. Second, increased noise may degrade reliability of a population response in coding the input signal, thus requiring more recruited neurons to ensure reliable response against noise distortion. The trade-offs between a reliable neural code and limited available energy result in an optimal number of neurons in maximal information transmission and minimal energy cost for a given noise level. Here, introducing the bistable model makes it feasible to analyze directly the input-output response function to the signals in noise environment, and provide a general solution for the energy-constrained efficient information transmission process. Since the bistable state describes the action potential initiation process in HH systems, the results presented here can be applied to all types of excitable neuronal models and real neurons. In addition, although our work focused on the effects of system size on energy efficiency, it could be extended to include the effects of spike correlation, stimulus and noise distribution on the energy efficiency, based on the recent progress on suprathreshold stochastic resonance^14^,^27^,^41^. Our analysis considered in detail the contribution of noise to information transmission regarding sub- and super-threshold stochastic resonance, while this could not have been derived from pure binary neurons in previous studies^22^.

The model presented is not complex enough to include details of ionic channel properties contributing to an energy efficient spiking process. Other recent research has focused on this issue and found^5^,^7^,^8^,^9^, ^23^,^25^ that there may exist an optimal number of ionic channels^19^ or an appropriate ratio of sodium to potassium channel density to generate energy efficient action potentials^5^,^9^,^23^,^25^. Therefore, for mature neurons with stabilized ionic channel densities and kinetics, in stable environmental conditions, our model rather considered a more general situation of a network case in the presence of noise, and proved that the coding energy cost per unit information goes through a global minimum with an optimal neuronal number depending on given noise intensity. In addition, the implications of achieved results with the dynamical model used here may be limited by it having fixed unit energy per spike. There is an additional possibility as reported by a few experimental and computational reports that the energy efficiency per spike may vary with stimuli conditions, which may add additional capacity of neural coding efficiency^42^,^43^,^44^. This would be worth further examination with a more realistic Hodgkin-Huxley neuronal model with proper ionic channels.

To summarize, in this paper, we examined the energy efficiency of an information coding process based on a neuronal array network composed of N simple bistable neurons and a CD detector neuron. We have provided an analytical solution that quantifies the relationships among the energy cost per neuron, mutual information, noise intensity, signal intensity and frequency, and neuronal number required in the circuit for effective information transmission. The novel result obtained here reveals a general rule of energetics related to population coding that there exists an optimal number of neurons in the network necessary for maximal information coding with minimal energy cost, and the optimum depends on the noise intensity, input pulse strength and frequency. The results reflect general mechanisms for sensory coding processes, which may give insight into energy efficient brain communication and neural coding.

## Additional Information

**How to cite this article**: Yu, L. *et al.* Energy-efficient population coding constrains network size of a neuronal array system. *Sci. Rep.* **6**, 19369; doi: 10.1038/srep19369 (2016).
